# Asiatic Cholera

**Published:** 1848-03

**Authors:** 


### ASIATIC CHOLERA.

The last advices from Europe, speak of the appearance of cholera on the borders of Prussia, but it is doubtful whether it has progressed so far. We do not discover, in fact, any certain information of its advance westwardly, nor does there seem to be so much excitement on the subject, so far as we can judge by the English and French journals.

